# Divergent Thinking Abilities in Frontotemporal Dementia: A Mini-Review

**DOI:** 10.3389/fpsyg.2021.652543

**Published:** 2021-04-16

**Authors:** Giulia Fusi, Maura Crepaldi, Laura Colautti, Massimiliano Palmiero, Alessandro Antonietti, Luca Rozzini, Maria Luisa Rusconi

**Affiliations:** ^1^Department of Clinical and Experimental Sciences, University of Brescia, Brescia, Italy; ^2^Department of Human and Social Sciences, University of Bergamo, Bergamo, Italy; ^3^Department of Psychology, Catholic University of the Sacred Heart, Milan, Italy

**Keywords:** divergent thinking, review, frontotemporal dementia, semantic memory, creativity

## Abstract

A large number of studies, including single case and case series studies, have shown that patients with different types of frontotemporal dementia (FTD) are characterized by the emergence of artistic abilities. This led to the hypothesis of enhanced creative thinking skills as a function of these pathological conditions. However, in the last years, it has been argued that these brain pathologies lead only to an augmented “drive to produce” rather than to the emergence of creativity. Moreover, only a few studies analyzed specific creative skills, such as divergent thinking (DT), by standardized tests. This Mini-Review aimed to examine the extent to which DT abilities are preserved in patients affected by FTD. Results showed that DT abilities (both verbal and figural) are altered in different ways according to the specific anatomical and functional changes associated with the diverse forms of FTD. On the one hand, patients affected by the behavioral form of FTD can produce many ideas because of unimpaired access to memory stores (i.e., episodic and semantic), but are not able to recombine flexibly the information to produce original ideas because of damages in the pre-frontal cortex. On the other hand, patients affected by the semantic variant are impaired also in terms of fluency because of the degradation of their semantic memory store. Potential implications, limitations, and future research directions are discussed.

## Introduction

Frontotemporal lobar degeneration (FTLD) represents a group of neurodegenerative brain diseases characterized by a fairly focal onset of degeneration of the frontal and/or anterior temporal lobes. One of the clinical entities associated with FTLD is frontotemporal dementia (FTD), a neurodegenerative disorder that expresses itself with different clinical presentations (Gossye et al., 2019). In particular, corresponding to the focal pattern of anatomical alterations, patients with FTD develop specific focal cognitive and behavioral deficits, which have led to evidence of several variants of FTD (Viskontas and Miller, 2007; Gorno-Tempini et al., 2011; see Table 1 for more details).

**Table 1:** Summary of anatomic, cognitive, and emotional/behavioral alterations observed in the FTD variants.

**FTD variant**	**Anatomical alterations**	**Cognitive and behavioral alterations**
Behavioral FTD	Atrophy in the frontal and temporal lobes, the insula and the anterior cingulate cortex (ACC), with the earliest involvement of frontal paralimbic cortices and insula (Meeter et al., 2017).	Remarkable change in personality with prominent apathy and disinhibition, accompanied by a lack of empathy and insight, stereotypical behaviors and changes in eating habits, obsessive-compulsive behaviors. Executive dysfunctions and altered social cognition are also observed yet with relative preservation of other cognitive areas such as visuospatial function and memory in the early stages (Musa et al., 2020).
Semantic variant of primary progressive aphasia	Asymmetrical (commonly left- sided) antero-inferior temporal lobe atrophy, hypometabolism or hypoperfusion	Anomia, impaired object knowledge, particularly for low- frequency or low-familiarity items, progressive loss of word meaning and impaired single words comprehension but with preserved fluency and repetition. Possible surface dyslexia and dysgraphia (Gorno-Tempini et al., 2011).
Non-fluent/agrammatic primary progressive aphasia	Predominantly left-sided inferior fronto-insular atrophy, hypometabolism or hypoperfusion	Characterized by agrammatism and/or apraxia of speech with progressive uncertainties of speech that in turn progress to mutism. Impaired comprehension of syntactically complex sentences, but with spared object knowledge and word comprehension (Gorno-Tempini et al., 2011).
Logopenic primary progressive aphasia	Left temporoparietal junction, posterior perisylvian or parietal atrophy, hypometabolism or hypoperfusion	Impaired single-word retrieval in spontaneous speech and naming and impaired repetition of sentences and phrases, possible phonological errors in spontaneous speech and naming but with spared single-word comprehension and object knowledge, motor speech and absence of frank agrammatism (Gorno-Tempini et al., 2011).

Interestingly, artistic creativity (i.e., poetry, paintings, etc.) can be expressed by some exceptional cases of patients affected by neurodegenerative diseases (Palmiero et al., 2012; de Souza et al., 2014), including specific variants of FTD (see Geser et al., 2021 and Miller and Miller, 2013 for a review). However, this “de novo” creative production is not equally observed in all FTD variants. Recent works suggest that these productions, in this type of patients, are rare (Abraham, 2019), and the idea that their artistic expressions are the result of the need for some form of communication (Zaidel, 2014), of a general “drive to produce” (Canesi et al., 2012), or of a “pseudo-creative production” triggered by their cognitive or behavioral characteristics such as perseveration or disinhibition (De Souza et al., 2010; de Souza et al., 2014), emerged. In addition, previous research assessed mostly patients' artistic productions instead of their creative cognition skills by standardized tests such as divergent thinking (DT) tasks. This means that the extent to which FTD patients are capable to express actual creativity has to be still demonstrated.

DT is not a synonym of creativity (Piffer, 2012; Runco and Jaeger, 2012), but it was assumed to lie at the heart of creative thinking (Runco and Acar, 2012; Jauk, 2019). DT relies on the ability to answer in a fluent (produce many relevant responses), flexible (consider different categories), original (produce unique ideas), and elaborate (embellish ideas with details) way to open-ended questions (Guilford, 1956). It has been recognized as a measure of the “creative potential” (Runco and Acar, 2012) and as a valid indicator of real-world creative achievements (Jauk et al., 2014). In this vein, the assessment of DT skills in FTD patients, who are characterized by specific anatomical and behavioral alterations, might be useful to better understand the neural substrates of creative cognition (Ovando-Tellez et al., 2019).

The aim of this mini-review is to summarize the main empirical findings regarding DT abilities in different FTD variants, highlighting literature gaps and providing some suggestions for future studies.

## Methods

The literature search of available sources describing DT abilities in patients with a diagnosis of FTD was conducted on PubMed, Google Scholar, and Scopus with the following keywords: “frontotemporal dementia AND divergent thinking,” “frontotemporal dementia AND creativity,” “primary progressive aphasia AND divergent thinking,” and “primary progressive aphasia AND creativity.” Only full-text journal article published in English that employed specific tasks or batteries recognized as standardized methods to address DT were considered (see Table 2).

**Table 2:** Studies exploring DT abilities in FTD patients: characteristics, provided data, and results.

**References**	***N* patients and diagnosis**	**Neuroimaging**	**DT measures**	**Material**	**DT indexes and scoring procedure**	**Results**
1. Hart and Wade (2006)	19 early DAT and 4 FTD; that were treated as a unique “Dementia group” (Mean age = 70.5 ± 8.4); HC (12, Mean age = 70.4, ± 5.9).	Np	Two complex fluency tasks (CFT): adapted AUT and Possible Jobs	Verbal	Not specified Only a total score of the two tasks were provided	All patients were impaired in every fluency task but AUT and Possible Jobs were more sensitive to early cognitive decline. The analyses were also run without FTD patients and the results were the same.
2. Rankin et al. (2007)	18 FTLD: 9 FTD (Mean age = 57.00, ± 5.96) and 9 SD (Mean age = 63.67, ± 6.87); 16 AD (Mean age = 71.00, ± 11.24); 15 HC (Mean age = 66.73, ± 9.37).	Np	TTCT (picture completion task only).	Figural	Fluency, Originality, Elaboration; Resistance to premature closure. + checklist of creative strengths	svPPA patients performed very poorly on standardized testing of non-verbal DT task (in all DT indexes: fluency, originality and elaboration). FTD failed to resist to premature closure, while AD performance is not distinguishable from HC.
3. De Souza et al. (2010)	17 bvFTD (Mean age = 71.1, ± 9.13). They showed a history of progressive decline in social interpersonal behavior, with emotional blunting and loss of insight. 17 HC (Mean age = 67.6, ± 6.71).	SPECT at rest: bvFTD patients showed a large bilateral frontal and temporal hypoperfusion	TTCT	Verbal, Figural	Total scores for verbal vs. figural DT. For each category fluency, flexibility and originality scores were calculated.	The bvFTD performed worse in all the DT tasks with respect to HC. Behavioral: The global TTCT score was correlated with general cognitive functioning and with frontal functions measures and batteries. Imaging: in bvFTD patients, positive correlations were mostly found between creativity tests (global, verbal and visual) and rostral pre-frontal cortex perfusion.
4. Ruggiero et al. (2019)	11 FTD (Mean age = 70, ± 4.83; 8bvFTD, 3svPPA) considered as a unique group. 15 HC (Mean age = 61.87, ± 8.55).	Np	DTT (Williams, 1994; Ruggiero et al., 2018).	Figural, verbal (only title)	Fluidity, flexibility, originality, processing	FTD patients showed a decreased performance in all DT subscores (except for the processing subscore).
5. Paulin et al. (2020)	15 probable bvFTD (Mean age = 62.9 ± 7.2). 10 svPPA (Mean age = 64.9 ± 6.2) characterized by marked language disturbances manifesting in impaired naming and comprehension, in the context of intact phonology and fluency of speech. 20 HC (Mean age = 66.8 ± 7.8).	bvFTD: prominent bilateral PFC and anterior temporal atrophy. svPPA: the majority (*n* = 7) displayed the classic left-lateralized presentation, beginning in the left anterior temporal lobe and evolving to a left-predominant, yet bilateral pattern, while three cases showed the less prevalent right-sided SD.	AUT	Verbal	Responses were scored in line with standard scoring methods (Barbot et al., 2019) focusing on three main outcome measures: Fluency, Originality, and Flexibility.	A decrease in all indexes (fluency, flexibility, originality) compared to controls were observed, with also a greater decrease in fluency in svPPA patients. In svPPA: the fluency index correlated robustly to the ability to make links between semantic concepts. Originality was associated with measures of semantic naming and comprehension. In bvFTD: the originality of the responses correlated with letter fluency and response inhibition.

*DAT, Dementia of Alzheimer's type; FTLD, Frontotemporal lobar degeneration; svPPA, semantic variant of primary progressive aphasia; AD, Alzheimer Disease; bvFTD, behavioral variant frontotemporal dementia; HC, Healthy Control; AUT, Alternative Uses Task; TTCT, Torrance Test of Creative Thinking; DTT, Divergent Thinking Test; Np, Not provided*.

## Result: Divergent Thinking in FTD Patients: State of the Art

All the selected studies showed a general decline in DT abilities. The following subsections summarize the main hypotheses proposed by the authors by subdividing them according to the considered FTD variant.

### FTD Behavioral Variant

Patients with the behavioral variant of FTD, characterized primarily by frontal alterations and significant behavioral changes such as apathy and disinhibition, stereotyped behaviors, and prominent dysfunction in executive and control functions, show a decline in both figural (Hart and Wade, 2006; De Souza et al., 2010; Ruggiero et al., 2019) and verbal (Rankin et al., 2007; De Souza et al., 2010; Paulin et al., 2020) DT. Hart and Wade (2006) revealed that a group of patients affected by dementia (i.e., patients with Alzheimer Disease – AD and behavioral variant FTD) scored low in terms of verbal fluency (number of relevant ideas) in both the Alternate Uses Task (asking to write down as many different uses for different objects) and Possible Jobs test (asking to list jobs that might be indicated by specific symbols or emblems). According to the authors, the decline of fluency in these patients might be explained by an executive dysfunction, which would reduce access to an intact semantic memory store. De Souza et al. (2010) evaluated patients' performances by considering the correlations between DT and both behavioral (executive functions tasks) and neuroimaging (cerebral perfusion) data. Results showed that DT scores assessed by the Torrance Test of Creative Thinking (TTCT) were correlated to patients' performance on the executive functions tests and, particularly, to fluency and flexibility, but not to attentional scores. Authors claimed that higher levels of frontal function impairment correlate to lower levels of divergent thinking. Moreover, authors suggested that proper functioning of the frontal poles supports DT, highlighting the role of top-down processes as pivotal for creative cognition. Accordingly, Ruggiero et al. Ruggiero et al. (2019), who evaluated a mixed FTD group (combining behavioral and semantic variants), suggested that poor creative abilities in these patients might be due to the alteration of the frontal lobes, which represent the main connection between the temporal and parietal lobes where knowledge and concepts are stored. Moreover, Paulin et al. (2020) found that patients with the behavioral variant showed impaired verbal DT in terms of fluency, flexibility, and originality as compared with healthy controls. In addition, the authors showed that the left middle frontal gyrus was strongly correlated to DT performance, confirming the role of frontal lobes in the creative process. Finally, Rankin et al. (2007) found no significant difference between patients affected by the behavioral variant FTD and healthy controls in the figural part of the TTCT, except for the resistance to premature closure (i.e., when incomplete figures are not closed by the quickest route), as if figural DT was impaired to a lesser extent.

### Semantic Variant of Primary Progressive Aphasia

Rankin et al. (2007) evaluated a cohort of patients with the semantic variant, characterized primarily by a selective degradation of conceptual knowledge, who showed a strong decline in figural DT. According to the authors, the preservation of semantic abilities may be somewhat necessary for conceptual flexibility and for moving beyond a concrete representation of the immediate visual stimuli and to perform some form of conceptual associations. Moreover, Paulin et al. (2020) highlighted that these patients were even less fluent than patients affected by the behavioral variant, in a verbal DT task. They showed also that the fluency index correlated robustly to a measure of semantic association which measures the ability to make links between semantic concepts; therefore, they hypothesized that the progressive deterioration of semantic knowledge in these patients precludes not only access to appropriate semantic constructs, but also impairs the capacity to draw novel associations between concepts in support of DT. In other words, semantic memory would be a necessary scaffold for a host of flexible expressions of creative cognition.

### Nonfluent and Logopenic Variant of Primary Progressive Aphasia (PPA)

No data are available.

## Discussion: The Interplay Between Memory Systems and Cognitive Control: What FTD Patients Can Tell us?

### The Role of Semantic Memory and the Default Mode Network

Creative cognition is hypothesized to rely on both episodic and semantic memory processes, even if it goes beyond them. Researchers widely agree on the fact that new ideas do not come from scratch but arise from meaningful variations and recombination of available knowledge (see Benedek and Fink, 2019 for a review). In particular, hippocampus, a brain structure which is involved in episodic memory, plays a key role in DT (Sheldon et al., 2011; Beaty et al., 2016). Besides, a growing amount of studies also support the role of semantic memory in creative cognition. Semantic memory is described as the store of concepts and facts (Budson and Price, 2005; McRae and Jones, 2013) and it is believed to be necessary for the ability to associate and connect useful ideas, especially if they are distant from each other (Mednick, 1962). In this direction, according to Mednick's theory, creative individuals would be characterized by a more flexible organization of words and concepts in their semantic memory, which allows them to activate and combine remote ideas. This claim has been applied to creativity in terms of “semantic distance”: this theoretical notion assumes that the farther one moves from a concept in the semantic space, the higher is the probability to generate a creative idea. Actually, recent studies confirmed that highly creative individuals are faster in generating associative responses between distant concepts, tend to provide higher percentages of unique associative responses (Kenett et al., 2014), show more associative links in their semantic network (Rossmann and Fink, 2010), as well as a more flexible semantic memory structure (i.e., higher connectivity, shorter distances between concepts, lower modularity; see Benedek et al., 2017; Kenett et al., 2018; Kenett and Faust, 2019; Beaty and Kenett, 2020; He et al., 2020). Moreover, associative abilities have been strictly related to creative cognition (Benedek et al., 2012; Beaty et al., 2014) and seem to mediate the relationship between the semantic memory individual structure and verbal creativity skills as if the spread of information in the semantic memory store could facilitate verbal creative thinking by associative abilities (He et al., 2020).

Summing up, there is evidence that remote elements of knowledge might be greatly interconnected in highly creative individuals and that a more flexible and efficient semantic memory structure and higher associative abilities are associated with creative performances. Moreover, Beaty et al. (2020) proved dissociable contributions of episodic and semantic memory processes to creative cognition, suggesting that distinct regions within the default mode network (DMN) that is usually associated with spontaneous and self-generated thought, support specific memory-related processes during DT. In addition, researchers confirmed the involvement of both the Semantic Control Network (SCN, Noonan et al., 2013), which is considered pivotal for flexible retrieval of stored knowledge (Cogdell-Brooke et al., 2020) and the DMN (Beaty et al., 2016, 2018) during DT tasks, highlighting their pivotal role in creative cognition, even if their specific interaction remains to be clarified (Ovando-Tellez et al., 2019).

In this light, it is not surprising to find that a deficit or an alteration of the semantic memory structure or content, such as the one that occurs in patients affected by the semantic variant of PPA, can cause a decline in the ability to generate several, novel, useful, and appropriate ideas. Indeed, these patients showed difficulty in providing original and elaborated solutions, probably because of their deficit in word finding and in the understanding of their meanings. Moreover, according to the degradation of their semantic system, patients with the semantic variant provide even fewer creative solutions than patients with other variants such as the behavioral one (Paulin et al., 2020). Additionally, the specific alteration of the semantic system seems to disrupt both verbal (Hart and Wade, 2006; Paulin et al., 2020) and figural DT (Rankin et al., 2007). Thus, an intact semantic store and the ability to access it flexibly (Hass, 2017; Forthmann et al., 2019) appear to be key requirements for accessing information that needs to be recombined to generate diverse and original ideas. Finally, it can be observed that there is a lack of standardized assessment on patients with different forms of primary progressive aphasia, such as the non-fluent and logopenic variants, which are characterized by a sparing of semantic and episodic memory stores in the early phase of the disease (Montembeault et al., 2018). This would help to better disentangle the major role of more general language abilities from that of semantic memory structure and functioning.

### The Role of Cognitive Control and the Executive Network

The generation of novel ideas depends not only on the ability to retrieve known information from episodic and semantic memory, but involves also the interaction between multiple control functions, which are related to the functioning of the frontal lobes (Benedek et al., 2014). Higher creative abilities have been correlated to the ability to overcome knowledge constraints imposed by the semantic knowledge structure (Abraham et al., 2012), to inhibit prepotent (Edl et al., 2014) and automatic responses (Gupta et al., 2012), to update and integrate old and new information (Zabelina et al., 2019), to use flexible cognitive control (Zabelina and Robinson, 2010), depending on the goals and the characteristics of a given task (Chrysikou et al., 2013), and to conduct a “goal-directed memory retrieval” (i.e., “the ability to strategically search episodic and semantic memory for task-relevant information”: Beaty et al., 2019, p. 22). Notably, these findings are also consistent with neuroimaging reviews and meta-analyses (i.e., Gonen-Yaacovi et al., 2013; Boccia et al., 2015; Wu et al., 2015), neuromodulation data (i.e., Colombo et al., 2015) and with clinical findings (Abraham et al., 2012; de Souza et al., 2014): all together these studies support the pivotal role of frontal regions and specifically of the executive network (EN, Beaty et al., 2016; Ovando-Tellez et al., 2019). Furthermore, an interplay between the DMN, which supports idea generation and association, and the EN, which is involved in guiding, constraining, and adapting DN processes to meet creative task goals (Beaty et al., 2019) is considered pivotal during the performance of DT tasks (Beaty et al., 2016), even in older people (Adnan et al., 2019). Thus, atrophy and/or impaired brain metabolism and perfusion in frontal areas, which characterize the behavioral variant of FTD, could selectively impair DT as a result of a deficit in top-down control processes, which prevent the inhibition of automatic and old ideas, to perform controlled retrieval in memory systems, and to integrate and associate remote information. This would clarify why these patients are more fluent during DT tasks than patients affected by the semantic variant of PPA, although they are less flexible and original than healthy controls. In other words, even if they access information, there is no strategic or top-down control, with the result that information is not used flexibly, and novel and appropriate ideas are not produced.

In conclusion, these results are consistent with the neurocognitive framework of creative cognition (Benedek and Fink, 2019) which assumed the key role of both the pre-frontal cortex (PFC), which drives attentional and cognitive control functions, and the hippocampus, which drives memory processes (both episodic and semantic). This means that a significant and specific decline in DT abilities can be caused by alterations exhibited by the different variants of FTD patients in the DMN, EN, or SMN. On the one hand, patients affected by the behavioral variant characterized by frontal and executive alterations, seem to be able to produce many ideas because of unimpaired access to both episodic and semantic memory stores, even though they are not able to recombine flexibly this information to produce original ideas. On the other hand, patients affected by the semantic variant, characterized by the degradation of the semantic memory store, are even more impaired than patients affected by the behavioral variant given that the semantic memory store is a necessary scaffold to perform DT tasks (Paulin et al., 2020).

## Limitations and Future Directions

Although the study of patients with neurological diseases has brought great progress in the understanding of the neural substrates and cognitive processes involved in DT, there are still too few studies to draw any final conclusions and, even more importantly, some methodological and theoretical issues need to be addressed.

Specifically, two out of five selected studies merged different groups of patients: Hart and Wade (2006) considered AD and FTD patients as a unique “dementia” group, whereas Ruggiero et al. (2019) merged patients affected by the behavioral variant of FTD and patients with the semantic variant of PPA as a unique FTD group. Then, even studies focused on specific categories of patients, such as the semantic variant PPA (Paulin et al., 2020), used patients with different atrophy patterns (left/right side presentation). Thus, although these studies led to some interesting hypotheses, the conclusions just drawn may be misleading because of these sampling issues. To this end, a more accurate selection, as well as an exhaustive behavioral, structural, and functional neuroimaging evaluation, can certainly lead to more reliable results in future studies and might be extremely useful not only in disentangling the role of both semantic memory structure and frontal functions, but also in deepening the notion about the existing relationships between DMN, SCN, and EN in creative cognition. Future studies might consider more systematically that verbal and figural/visuo-spatial abilities rely on both multi-componential and specific neural networks (Boccia et al., 2015), and the extent to which these abilities are selectively altered by different FTD variants. In this direction, future studies could also explore if different forms of DT (i.e., visual, musical, and motor - see Palmiero et al., 2019, 2020) can be used to bypass language deficits (i.e., agrammatism and impaired fluency) of patients with non-fluent and logopenic variants of primary progressive aphasia to assess their creative cognition abilities; they are indeed characterized by the sparing in semantic and episodic memory networks and, at least in part, in the control functions needed to accomplish this type of tasks. In this direction, future studies could also explore whether different forms of DT (i.e., visual, musical, and motor - see Palmiero et al., 2019, 2020) can be used to bypass the language deficits (i.e., agrammatism and impaired fluency) of patients with non-fluent and logopenic variants of primary progressive aphasia to assess their creative cognition abilities. Indeed, these patients are characterized by sparing, at least in the early stages, in the semantic and episodic memory networks and, in part, in the control functions necessary to perform these types of tasks and could therefore provide additional insight into the role of these systems in creative thinking abilities.

Finally, it is worth noting that recognizing the specific neurofunctional underpinnings (i.e., critical brain areas and cognitive functions) associated with DT through in-depth assessments of FTD patients could also have some important practical implications. In particular, this could help researchers to implement cognitive stimulation programs through DT tasks to improve cognitive functioning in both healthy older adults (see, e.g., Fusi et al., 2020b) and patients with various neurological or neurodegenerative diseases (Colautti et al., 2018; Ruggiero et al., 2019), especially in the early stages (Hart and Wade, 2006; Fusi et al., 2020a).

## Author Contributions

GF wrote the article. MC, LC, and MP were involved in the literature search, in the discussion of theoretical and methodological issues, and supported the writing procedures. AA, LR, and MR supervised all the writing process and the hypotheses advanced. All authors contributed to the article and approved the submitted version.

## Conflict of Interest

The authors declare that the research was conducted in the absence of any commercial or financial relationships that could be construed as a potential conflict of interest.
