# Erratum: Divergent Thinking Abilities in Frontotemporal Dementia: A Mini-Review

**DOI:** 10.3389/fpsyg.2021.749651

**Published:** 2021-09-02

**Authors:** Frontiers Production Office

**Affiliations:** Frontiers Media SA, Lausanne, Switzerland

**Keywords:** divergent thinking, review, frontotemporal dementia, semantic memory, creativity

There was an production error in the reference details for Palmiero et al., 2019.

Instead of “Palmiero, M., Giulianella, L., Guariglia, P., Boccia, M., D'Amico, S., and Piccardi, L. (2019). The dancers' visuo-spatial body map explains their enhanced divergence in the production of motor forms: evidence in the early development. Front. Psychol. 10:768. doi: 10.3389/fpsyg.2019.01274,” it should be “Palmiero, M., Giulianella, L., Guariglia, P., Boccia, M., D'Amico, S., and Piccardi, L. (2019). The dancers' visuospatial body map explains their enhanced divergence in the production of motor forms: evidence in the early development. Front. Psychol. 10:768. doi: 10.3389/fpsyg.2019.00768”.

The publisher apologizes for this mistake. The original version of this article has been updated.

## References

[B1] Palmiero M. Giulianella L. Guariglia P. Boccia M. D'Amico S. Piccardi L. (2019). The dancers' visuospatial body map explains their enhanced divergence in the production of motor forms: evidence in the early development. Front. Psychol. 10:768. 10.3389/fpsyg.2019.00768 31024403 PMC6467967

